# Breast cancer immunopeptidomes contain numerous shared tumor antigens

**DOI:** 10.1172/JCI166740

**Published:** 2024-01-02

**Authors:** Eralda Kina, Jean-Philippe Laverdure, Chantal Durette, Joël Lanoix, Mathieu Courcelles, Qingchuan Zhao, Anca Apavaloaei, Jean-David Larouche, Marie-Pierre Hardy, Krystel Vincent, Patrick Gendron, Leslie Hesnard, Catherine Thériault, Maria Virginia Ruiz Cuevas, Grégory Ehx, Pierre Thibault, Claude Perreault

**Affiliations:** 1 Institute for Research in Immunology and Cancer (IRIC), and; 2 Department of Medicine, University of Montreal, Montreal, Quebec, Canada.; 3 Laboratory of Hematology, GIGA-I3, University of Liege and CHU of Liège, Liege, Belgium.; 4 Department of Chemistry, University of Montreal, Montreal, Quebec, Canada.

**Keywords:** Immunology, Oncology, Antigen presentation, Breast cancer, Cancer immunotherapy

## Abstract

Hormone receptor–positive breast cancer (HR+) is immunologically cold and has not benefited from advances in immunotherapy. In contrast, subsets of triple-negative breast cancer (TNBC) display high leukocytic infiltration and respond to checkpoint blockade. CD8^+^ T cells, the main effectors of anticancer responses, recognize MHC I–associated peptides (MAPs). Our work aimed to characterize the repertoire of MAPs presented by HR+ and TNBC tumors. Using mass spectrometry, we identified 57,094 unique MAPs in 26 primary breast cancer samples. MAP source genes highly overlapped between both subtypes. We identified 25 tumor-specific antigens (TSAs) mainly deriving from aberrantly expressed regions. TSAs were most frequently identified in TNBC samples and were more shared among The Cancer Genome Atlas (TCGA) database TNBC than HR+ samples. In the TNBC cohort, the predicted number of TSAs positively correlated with leukocytic infiltration and overall survival, supporting their immunogenicity in vivo. We detected 49 tumor-associated antigens (TAAs), some of which derived from cancer-associated fibroblasts. Functional expansion of specific T cell assays confirmed the in vitro immunogenicity of several TSAs and TAAs. Our study identified attractive targets for cancer immunotherapy in both breast cancer subtypes. The higher prevalence of TSAs in TNBC tumors provides a rationale for their responsiveness to checkpoint blockade.

## Abstract 2

Proteogenomics of primary human breast cancer samples shows higher abundance of tumor-specific antigens in triple-negative tumors than hormone-receptor-positive breast cancers and provides targets for immunotherapy.

## Graphical abstract

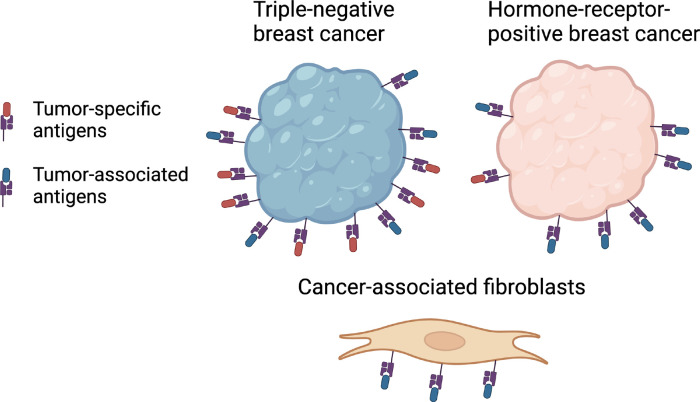

## Introduction

According to recent global statistics, breast cancer is now the most prevalent cancer worldwide, outranking lung cancer (1). Despite therapeutic advances in the last decades, metastatic breast cancer remains incurable. From an immunological standpoint, breast cancer tumors fall into two main categories (2). Hormone receptor–positive breast cancer (HR+) is considered an immunologically “cold” cancer and has not benefited from recent advances in immunotherapy (3). In contrast, subsets of the triple-negative breast cancer (TNBC) subtype are immunologically “hot,” as evidenced by high levels of leukocytic infiltration and responsiveness to immune checkpoint blockade (ICB) (4, 5). In fact, following KEYNOTE-355, the addition of ICB to chemotherapy has become the new standard of care for TNBC tumors with high programmed death-ligand 1 (PD-L1) expression (6). High tumor mutational burden (TMB) has previously been used as a biomarker for response to ICB in multiple cancers (7). However, it is only present in 5% of breast cancer tumors and is not correlated with CD8^+^ T cell infiltration (7, 8). Therefore, the cause of the differential immunogenicity of HR+ and TNBC tumors remains elusive. As CD8^+^ T cells recognize MHC I–associated peptides (MAPs), we hypothesized that differences in the MAP repertoire of HR+ and TNBC tumors might explain this discrepancy.

In the present study, we focused on two broad groups of MAPs: tumor-specific antigens (TSAs) and overexpressed tumor-associated antigens (TAAs). TSAs are exclusively presented by tumor cells (9). Their absence in medullary thymic epithelial cells (mTECs) and extrathymic tissues means they cannot induce central or peripheral immune tolerance (9). Initial studies aiming at identifying TSAs focused on mutated TSAs (mTSAs) originating from cancer-specific nonsynonymous mutations. A few mTSAs have been validated by mass spectrometry (MS) in melanoma tumors (10). However, most mTSA predictions based on exome sequencing and MHC-binding algorithms were not validated by MS analyses, suggesting that they are much rarer than initially conjectured (11–13). In addition, the vast majority of mTSAs are private antigens. Aberrantly expressed TSAs (aeTSAs) are unmutated MAPs that arise from cancer-specific epigenetic changes and splicing aberrations (14). aeTSAs may originate from any genomic region (exons, introns, intergenic areas, etc.), and their key advantage is that they are highly shared between tumors (12, 15). In acute myeloid leukemia and ovarian cancer, aeTSAs thus far identified originated primarily from introns and intergenic regions (12, 15).

Because TAAs are not cancer specific, the majority are less immunogenic than TSAs. Nonetheless, MS has validated the presence of TAAs, and a few TAAs were shown to elicit biologically relevant antitumor responses in vivo (16–19). Features differentiating immunogenic from nonimmunogenic TAAs are a matter of speculation. They may hinge on TAA abundance (at the peptide level) in various normal tissues, including mTECs (20, 21). The term cancer-testis antigen (CTA) is commonly but loosely used in cancer immunology. This is unfortunate because some CTAs are immunogenic in humans (16). The confusion stems from the fact that the initial list of CTAs (22) was assembled before the era of big multiomic data. Hence, MS analyses revealed the presence in normal tissues of many MAPs labeled as CTAs (21, 23). Therefore, we categorize CTAs as aeTSAs if they are not expressed in any normal tissue (including mTECs) aside from the testis and as TAAs if expressed in one or many normal tissues (9).

Ternette and colleagues have demonstrated the feasibility of performing immunopeptidomic analyses on a few primary TNBC tumors (24). They identified a few MAPs more abundant in tumors than in the normal adjacent tissue, but did not fully characterize these antigens. Unexpectedly, given the frequency of breast cancer, we are unaware of other MS analyses on primary human breast cancer. Hence, we performed comprehensive proteogenomic analyses of 26 primary breast cancers to address this unmet need. The approach we used has 2 critical features. First, it is based on MS analyses rather than on predictions. Second, our approach is genome wide (rather than being limited to the exome). This feature is particularly relevant to the identification of TSAs. Our searches’ genome-wide scope enhances their breadth by allowing the identification of TSAs coded by any reading frame of all genomic regions.

## Results

### Global proteogenomic strategy for MAP identification in primary breast cancer samples.

We analyzed 26 primary breast cancer samples (14 HR+ and 12 TNBC) from untreated patients. MAPs were identified by MS analyses using a previously described proteogenomic approach (12, 14, 15). For each sample, we built personalized reference databases by in silico translation of RNA-Seq data. All MS databases included a canonical proteome (in-frame translation of protein-coding exons) used as is or combined with either (a) an endogenous retroelements (ERE) proteome, (b) a small RNA (smRNA) proteome, or (c) a cancer-specific proteome. The cancer-specific proteome contained RNA-Seq reads present in the tumor sample, but not in a collection of mTECs (Supplemental Figure 1A; supplemental material available online with this article; https://doi.org/10.1172/JCI166740DS1). Consistent with their critical role in the induction of central immune tolerance, mTECs express more genes than all other somatic cells (25). Therefore, the identification of cancer-specific MAPs deriving from sequences not expressed in mTECs is a pivotal feature of our approach, as it increases significantly the chances of identifying immunogenic targets (26).

### Canonical immunopeptidomes of HR+ and TNBC tumors are similar.

**Figure 1:**
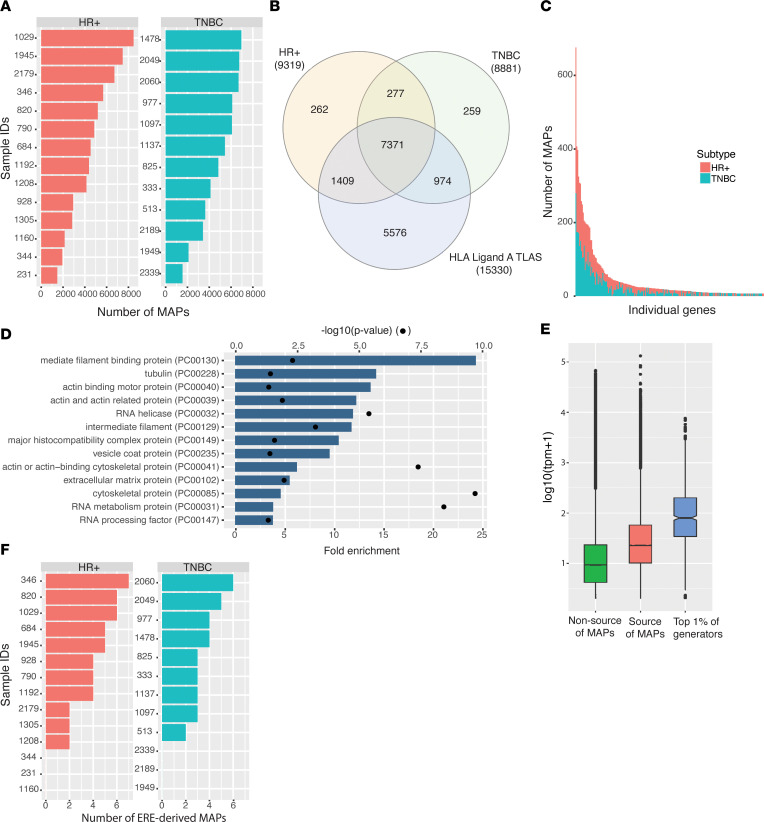
Canonical immunopeptidomes of HR+ and TNBC tumors. (**A**) Number of unique MAPs identified per sample (*n* = 26). (**B**) Venn diagram of source genes of MAPs in HR+ tumors, TNBC tumors, and normal tissues from the HLA Ligand Atlas. (**C**) Total number of MAPs per source gene for the top 1% of MAP generators. (**D**) Enrichment analysis of PANTHER protein classes for the top 1% of MAP generators (*n* = 242). (**E**) Expression of gene nonsources of MAPs, sources of MAPs, and top 1% of MAP generators (tpm > 1, ANOVA; *P* < 0.001). (**F**) Number of ERE-derived MAPs identified in HR+ and TNBC samples.

MAPs coded by the canonical reading frame of annotated protein-coding genes are collectively referred to as the canonical immunopeptidome. We identified a total of 57,094 canonical MAPs deriving from 10 552 protein-coding genes. The mean number of MAPs per tumor sample was 4,633, with no differences between HR+ and TNBC samples (*t* test, *P* > 0.05; Figure 1A). MAPs were presented by 53 different HLA alleles, with more than 20% presented by 2 common alleles: HLA-A*02:01 and HLA-B*18:01 (Supplemental Figure 1B). Consistent with a previous report (27), 62% of the transcriptome, defined as genes with more than 1 transcript per kilobase million (TPM), generated MAPs (Supplemental Figure 2A). A high proportion of source genes (72.5%, *n* = 7648) led to MAP generation in both HR+ and TNBC tumors (Figure 1B), and most of them (92%, *n* = 9754) were also reported as MAP source genes in normal tissues from the HLA Ligand Atlas (21).

Of the 798 canonical MAPs not listed in the HLA Ligand Atlas, 277 were found in HR+ and TNBC samples, 259 were unique to TNBC, and 262 to HR+ samples (Figure 1B). The 277 canonical MAPs shared by HR+ and TNBC samples were coded by a gene set enriched in extracellular matrix protein-coding genes (fold enrichment 7.59, *P* < 0.05). The 259 canonical MAPs found only in TNBC samples showed enrichment in the glycosyltransferase protein class (fold enrichment 5.58, *P* < 0.05). No particular enrichment was found for the 262 MAPs found only in HR+ samples.

Source genes did not uniformly contribute to the immunopeptidome. While 49% of the source genes from our data set generated 5 or fewer MAPs, 51% generated more than 5 MAPs, and 63 genes coded for more than 100 MAPs (Figure 1C and Supplemental Figure 2B). The top 1% of MAP generators were notably enriched in cytoskeletal and extracellular matrix protein classes (Figure 1D) and represented genes with higher expression than other immunopeptidome contributors (Figure 1E). Altogether, these results reveal a substantial overlap between the canonical immunopeptidomes of HR+ and TNBC samples and a conspicuous enrichment in MAPs derived from cytoskeletal and extracellular matrix proteins. The immunopeptidome projects at the cell surface a representation of proteins actively translated and degraded within the cells (28). Therefore, the overrepresentation of MAPs derived from cytoskeletal and extracellular matrix proteins is coherent with the crucial role of extracellular matrix remodeling in breast cancer tumorigenesis (29–31).

### The contribution of EREs and smRNAs to the noncanonical breast cancer immunopeptidome.

EREs and smRNAs are implicated in different steps in neoplastic transformation, including breast cancer (32, 33). Furthermore, EREs have already been shown to code for immunogenic MAPs in mice and humans (14, 34). Therefore, we specifically interrogated our personalized proteogenomic databases for the presence of ERE- and smRNA-coded MAPs in breast cancer. ERE-coding transcripts were retrieved from bulk RNA-Seq, whereas we used smRNA-Seq to build smRNA databases (Supplemental Figure 3, A and B).

We identified 75 ERE-derived MAPs, equally distributed between HR+ and TNBC samples (Figure 1F and Supplemental Table 3). ERE-derived MAPs mapped similarly to intronic and intergenic regions (Supplemental Figure 3C). The 3 main classes of EREs (e.g., long interspersed nuclear elements [LINE]; long terminal repeats [LTR]; short interspersed nuclear elements [SINE]) contributed similarly to the immunopeptidome of HR+ and TNBC samples (Supplemental Figure 3D). Only 9 of 22 ERE families led to MAP generation; the L1 family was the most important contributor (Supplemental Figure 3E). ERE subfamilies that generated MAPs were expressed at a higher level than EREs generating no MAPs (Supplemental Figure 3F). We identified only 3 smRNA-derived MAPs (Supplemental Table 3): 1 derived from a piwi-interacting RNA (piRNA) and 2 others from snRNAs. We conclude that, unlike smRNA-derived MAPs, ERE-derived MAPs are found in significant numbers in the immunopeptidome of breast cancer tumors.

In addition to our rigorous filtering steps (see Methods), we tested the correlation between predicted and observed retention times, a parameter considered a gold standard in the literature (35), to support our identifications’ validity. Both ERE- (*r* = 0.98, *P* < 0.001) and smRNA-derived (*r* = 0.98, *P* < 0.001) MAP retention times were highly correlated (Supplemental Figure 3H). Additionally, we evaluated the distribution of spectral angles as calculated with Prosit (TMT 2021 HCD), a software capable of predicting similarities between theoretical and observed spectra (Supplemental Figure 3, G and I). While the distribution for ERE-derived MAPs was similar to that for other MAPs (*f* test, *P* = 0.15), smRNA-derived MAPs showed even higher mean spectral angle scores (0.90) compared with other MAPs (0.84, *f* test, *P* = 0.001). Hence, our stringent filtering and validation steps support our identification of noncanonical MAPs.

### Identification of potential therapeutic targets: TAAs, aeTSAs, and mTSAs.

**Figure 2:**
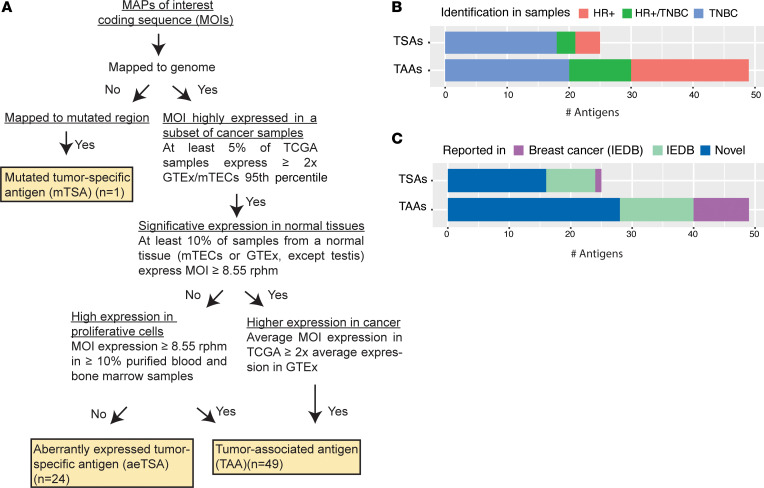
Identification of tumor antigens of interest. (**A**) Classification workflow for MOI. (**B**) Number of TSAs and TAAs identified in HR+ samples, TNBC samples, or both. (**C**) Proportion of TSAs and TAAs previously reported in the IEDB.

We next took advantage of our large immunopeptidomic data set to discover putative therapeutic breast cancer targets (Supplemental Table 4). MAPs of interest (MOIs) were classified as TAAs, aeTSAs, or mTSAs with the workflow outlined in Figure 2A. Overall, we identified 25 TSAs: 1 mTSA and 24 aeTSAs (Figure 2A). Identification of mTSAs is straightforward: they derive from mutated genomic sequences. The sole mTSA identified in our study originated from a rare nonsynonymous mutation in the deubiquitinase *OTUB1* gene not listed in the COSMIC database (36). The scarcity of mTSAs led us to ask whether genes frequently mutated in breast cancer were represented in the immunopeptidome. We found a slightly positive correlation between the frequency of mutations identified by The Cancer Genome Atlas (TCGA) consortium and the generation of MAPs in our data set (*P* < 0.001) (Supplemental Figure 4A). This means there was no negative bias against the representation of highly mutated genes in the breast cancer immunopeptidome. Hence, highly mutated genes generate MAPs, but these MAPs do not derive from the mutated region. This is consistent with the fact that MAPs preferentially originate from particular regions of MAP source proteins (MAP “hotspots”) (21, 27, 37). The most parsimonious explanation for the scarcity of mTSAs is that breast cancers harbor relatively few mutations and these mutations are not located in MAP hotspots.

Classification of unmutated MAPs as TAAs or aeTSAs is more complex and was based on comprehensive transcriptomic analyses of their expression in (a) breast cancer samples from TCGA (*n* = 1109), (b) 50 normal tissues from the Genotype-Tissue Expression (GTEx) project (https://www.gtexportal.org/home/) (5–150 samples per tissue), (c) mTECs (*n* = 11), and (d) purified blood and bone marrow samples (*n* = 4–16) (Figure 2A; see Methods, *MOI expression in tissues*). Blood and marrow cells are used as a surrogate for rapidly proliferating cells, since 90% of cells produced daily in humans are hematopoietic cells (38). It must be stressed that our expression profiling only considers the MAP-coding sequence, not the entire gene or genomic region. Since aberrations in RNA splicing commonly lead to the presence of protein isoforms only in cancer cells, MAPs derived from such cancer-specific isoforms are labeled as aeTSAs even if other isoforms are expressed in normal cells.

We considered only MAPs coded by transcripts overexpressed in at least 5% of TCGA breast cancer cohort samples. We assumed that antigens expressed in fewer samples had little therapeutic interest. MAPs whose expression was below 8.55 reads per hundred million (rphm) in all normal tissues except the testis were labeled as aeTSAs. As reported (12), we used 8.55 rphm as a threshold because expression below this level is associated with a very low probability of MAP generation in normal tissues. Notably, none of our aeTSAs are listed in the HLA Ligand Atlas of normal tissues and organs (21). Otherwise, MOIs with above threshold expression in one or more normal tissues were labeled as TAAs if overexpressed in neoplastic or highly proliferative tissues compared with normal nonhematopoietic tissues. This strategy led to the identification of 24 aeTSAs and 49 TAAs, most of which were not listed in the Immune Epitope Database (IEDB) (Figure 2, B and C, and Supplemental Table 5).

### TSAs are more abundant in TNBC than HR+ breast cancers.

**Figure 3:**
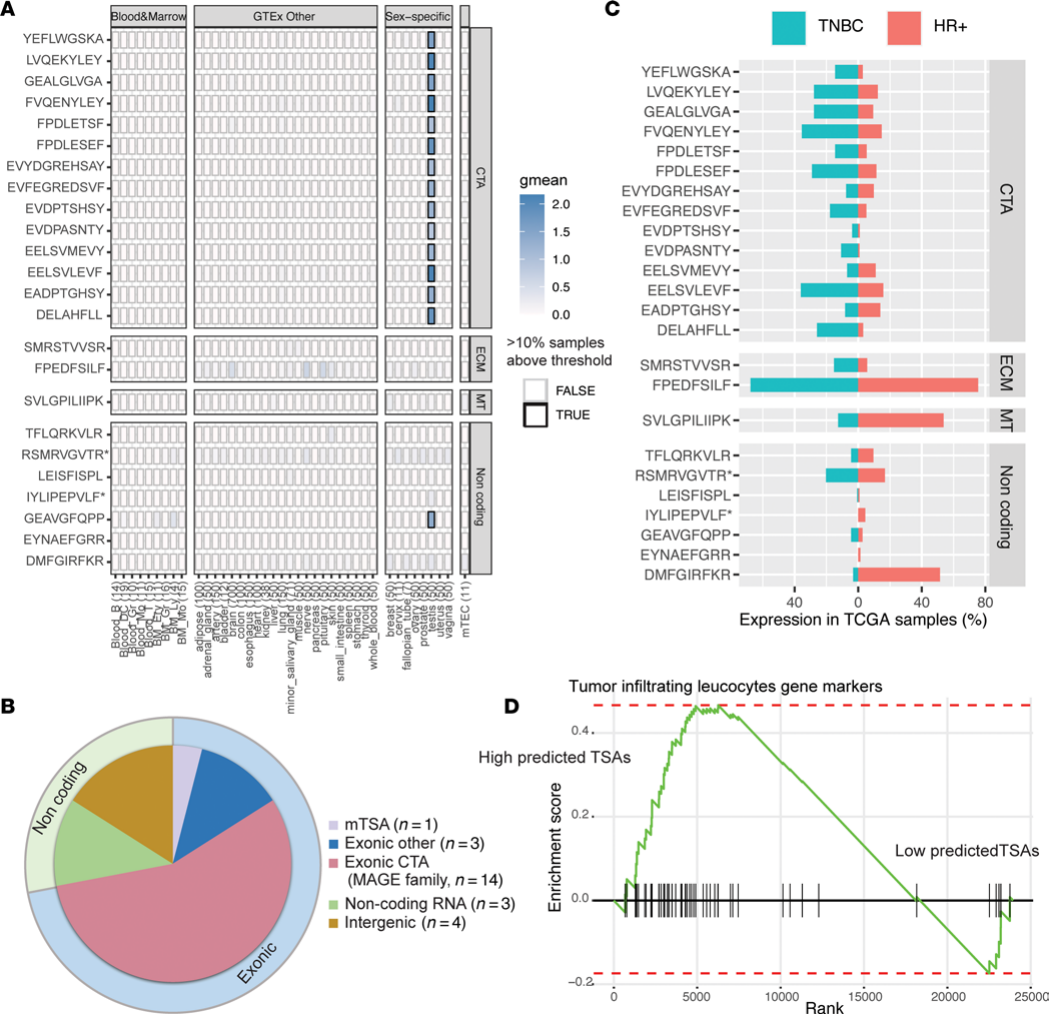
Identification of TSAs. (**A**) Expression heatmap of aeTSAs’ coding sequence in normal tissues (GTEx, mTECs, and bone marrow). Number of samples per tissue are shown in parentheses. Color intensity corresponds to average expression per tissue (mean log-transformed rphm). Bold boxes indicate that more than 10% of samples have an expression above 8.55 rphm. TSAs with an asterisk are also categorized as ERE-derived MAPs. ECM, extracellular matrix; MT, membrane transporter (ABCC11). (**B**) Genomic origin of identified TSAs. (**C**) Percentage of HR+ (*n* = 583) and TNBC (*n* = 158) tumors from TCGA-BRCA cohort with individual aeTSA expression of more than 2 rphm. (**D**) Enrichment analysis of tumor-infiltrating leucocyte gene markers in tumors with high levels (> median) of predicted aeTSAs (39). Normalized enrichment score = 1.59; adjusted *P* < 0.01.

Of the 24 aeTSAs, 17 were coded by canonical exons: 14 belonged to the MAGE family of CTAs, 2 to genes coding extracellular matrix components (*COL11A1*, *ITH6*), and 1 to a transmembrane protein-coding gene (*ABCC11*) (Figure 3 and Supplemental Table 5). Seven aeTSAs were derived from non–protein-coding regions, 2 of which overlapped EREs and can be classified as ERE-derived MAPs (Figure 3, A and B, and Supplemental Table 5). Most of our aeTSAs were found in TNBC samples (Figure 2B). We sought to evaluate whether this enriched identification in TNBC samples correlated with TSA expression in the TCGA cohort. The proportion of tumors expressing individual aeTSAs of the CTA class was superior in TNBC relative to HR+ tumors (19% versus 8%, *P* = 0.004) (Figure 3C). There was no significant difference in the distribution for the other categories of TSAs.

We next sought to evaluate whether aeTSA presentation would correlate with immune infiltration. An aeTSA was considered to be presented in a TCGA sample only when the MAP-coding transcript was expressed and the patient had an HLA allotype that could present this MAP (12). Next, we categorized breast cancer TCGA samples into 2 groups presenting high (above median) versus low (below median) numbers of aeTSAs. Then we performed a differential gene expression analysis between the 2 groups. A gene set enrichment analysis (GSEA) using gene markers of leukocytic infiltration described by Danaher et al. (39) showed enrichment of these genes in tumors with high TSA numbers (Figure 3D). This suggests that at least some TSAs are immunogenic in vivo. Additionally, we evaluated whether oncogenic pathways involved in immune escape (40) were enriched in samples with high or low levels of TSAs. To this end, we selected the corresponding hallmark gene sets from the Molecular Signature Database (41) and found enrichment in the PI3K pathway (normalized enrichment score = –1.48, *P* < 0.05; Supplemental Figure 4B) in tumors with low numbers of predicted TSAs. The PI3K pathway is implicated in tumorigenesis, progression, and resistance to treatment in breast cancers (42).

### TAAs are highly shared in breast cancer.

**Figure 4:**
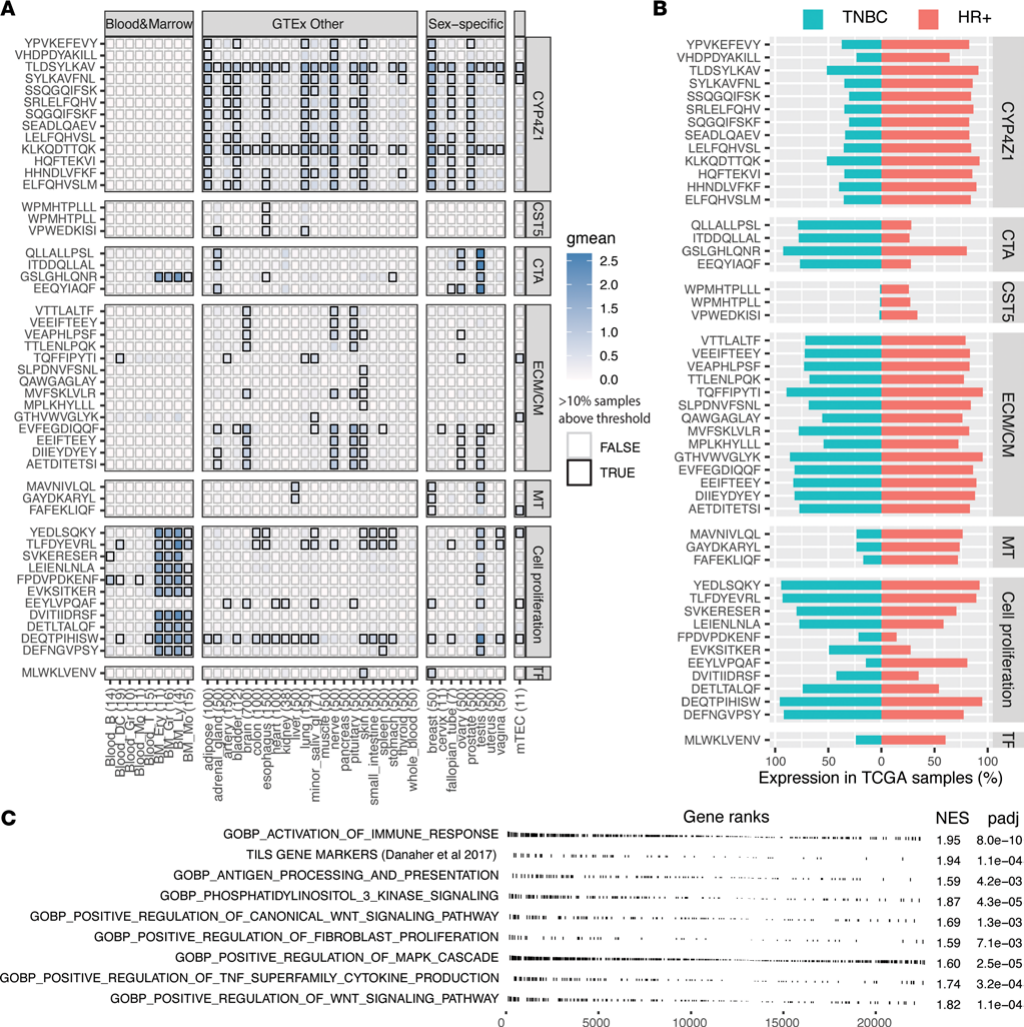
Identification of TAAs. (**A**) Expression heatmap of TAAs’ coding sequence in normal tissues (GTEx, mTECs, and bone marrow). Number of samples per tissue are shown in parentheses. Color intensity corresponds to average expression per tissue (mean log-transformed rphm). Bold boxes indicate that more than 10% of samples have an expression above 8.55 rphm. CM, cell migration; TF, transcription factor. (**B**) Percentage of HR+ (*n* = 583) and TNBC (*n* = 158) tumors from TCGA-BRCA cohort with individual TAA expression greater than 2 rphm. (**C**) GSEA in HR+ breast cancer tumors from the TCGA cohort with high levels (>median) of predicted TAAs.

**Figure 5:**
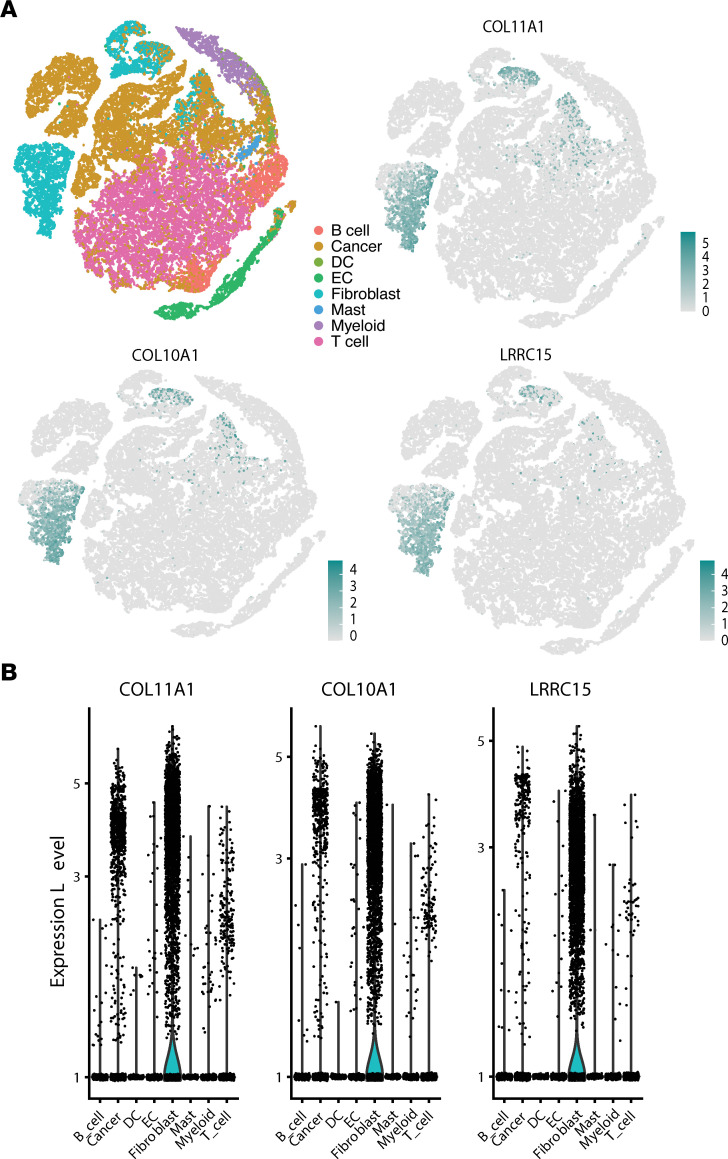
CAF-derived TAAs. (**A**) t-Distributed stochastic neighbor embedding (t-SNE) showing that *COL11A1*, *COL10A1*, and *LRRC15* are mainly expressed in fibroblasts in 14 primary breast cancer samples from Qian et al. (44). (**B**) *COL11A1*, *COL10A*1, and *LRRC1*5 are overexpressed in fibroblasts compared with other cell types found in breast cancer samples (ANOVA; *P* < 0.05).

We identified 49 TAAs, of which 48 originated from canonical protein-coding regions (Figure 4A and Supplemental Table 5). These antigens were highly shared in both HR+ and TNBC tumors (Figure 4B). The largest group of TAAs (*n* = 14) derived from genes reported as markers of cancer-associated fibroblasts (CAFs) (*COL11A1*, *COL10A1*, *LRRC15*) (43). These genes are implicated in extracellular matrix production and cell migration. To assess their most likely cell of origin, we evaluated their expression level in a single cell data set from Qian et al. (44) comprising 14 breast cancer tumors (Figure 5A). All 3 genes had significantly higher expression in tumor fibroblasts than in other cell subsets, including cancer cells (Figure 5B; ANOVA, *P* < 0.05). We identified 2 other large groups of TAAs. Thirteen TAAs were derived from *CYP4Z1,* which is implicated in many cancer types and elicits autoantibodies in breast cancer patients (45). Eleven TAAs were associated with cell proliferation (Figure 4B); they were expressed at low levels in mature epithelial and blood cells, but at higher levels in bone marrow progenitor cells (Figure 4A).

Again, we predicted the number of TAAs per tumor in the TCGA data set using the same criteria as for aeTSA: expression of the MAP-coding sequence and presence of a relevant HLA allotype (Figure 4C and Supplemental Figure 4C). HR+ and TNBC tumors with a high level of predicted TAAs showed enrichment in immune activation and immunosuppressive pathways, namely the PI3K/mTOR, Wnt/B-catenin, and MAPK pathways. In addition, tumors with numerous TAAs showed enrichment in markers of fibroblast proliferation. These findings suggest that in the presence of multiple TAAs, antitumor immune responses are mitigated by the activation of immunosuppressive pathways and the accumulation of CAFs.

### Immunogenicity of TSAs and TAAs.

**Figure 6:**
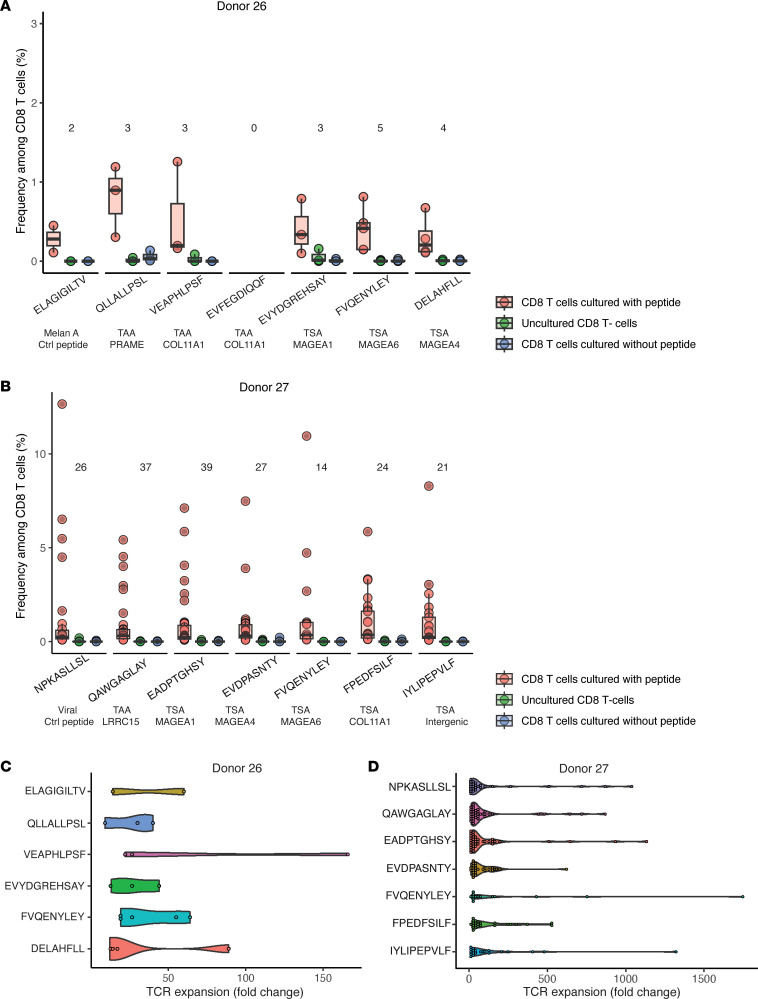
In vitro immunogenicity of TSAs and TAAs. FEST assays were conducted after 20 days of stimulation with autologous T cell–depleted PBMCs pulsed with individual peptides. (**A** and **B**) Frequency of specific clonotype expansion in donors 26 and 27. The number of significantly expanded clonotypes is indicated above each peptide. Source genes are indicated below the peptide sequences of TAAs and TSAs. (**C** and **D**) Expansion of antigen-responsive T cell clonotypes compared with unpulsed CD8^+^ T cells in donors 26 and 27.

In order to evaluate the immunogenic potential of identified antigens, we performed functional expansion of specific T cell (FEST) assays, which involve TCR Vβ CDR3 sequencing of CD8^+^ T cells stimulated or not with individual synthetic peptides (see Methods). FEST assays estimate 2 features of antigen-specific T cells: their proliferative capacity and the diversity of their TCR repertoire. Therefore, we reasoned that FEST assays were particularly suitable to evaluate the functionality of TAA- and aeTSA-specific T cells. We tested 11 different antigens (7 TSAs and 4 TAAs) presented by HLA allotypes found in 2 healthy blood donors (Figure 6). Positive controls comprised the immunogenic modified MelanA-derived MAP ELAGIGILTV and respiratory syncytial virus RSV-NL9 peptide NPKASLLSL.

Significative expansion of TCR Vβ clonotypes (from 3 to 39 clonotypes) was detected following priming with each of the 7 TSAs. These TSAs were derived from genes of the MAGEA family (*n* = 5), COL11A1 (*n* = 1), and an intergenic region overlapping an ERE (*n* = 1) (Figure 6). Three of the 4 tested TAAs led to a significative expansion of 2 to 37 clonotypes. Two immunogenic TAAs were derived from LRRC15 and COL11A1 (2 genes expressed in CAFs), and 1 was derived from PRAME. The polyclonality of TCR clonotypes recognizing our TAAs and TSAs is relevant, since the number of neoantigen-specific TCR clonotypes correlates with antitumor responses in patients treated with anti-PD1 (46). Moreover, the significant expansion of TAA- and TSA-responsive T cells (5-fold to 1,747-fold) is notable because T cells’ proliferative capacity is the first effector function lost when they become exhausted or anergic (47). We conclude that healthy subjects’ repertoire contains polyclonal and functional TAA- and TSA-specific T cells.

### Presentation of numerous TSAs improves overall survival in TNBC.

We next sought to evaluate whether the number of presented TAAs and aeTSAs correlated with the overall survival of TCGA patients. We considered that an antigen was presented in a tumor when both the MAP-coding transcript and an appropriate HLA allotype were expressed (12). Patients were divided into 2 categories: high number of presented antigens (> median) and low number of presented antigens (≤ median).

**Figure 7:**
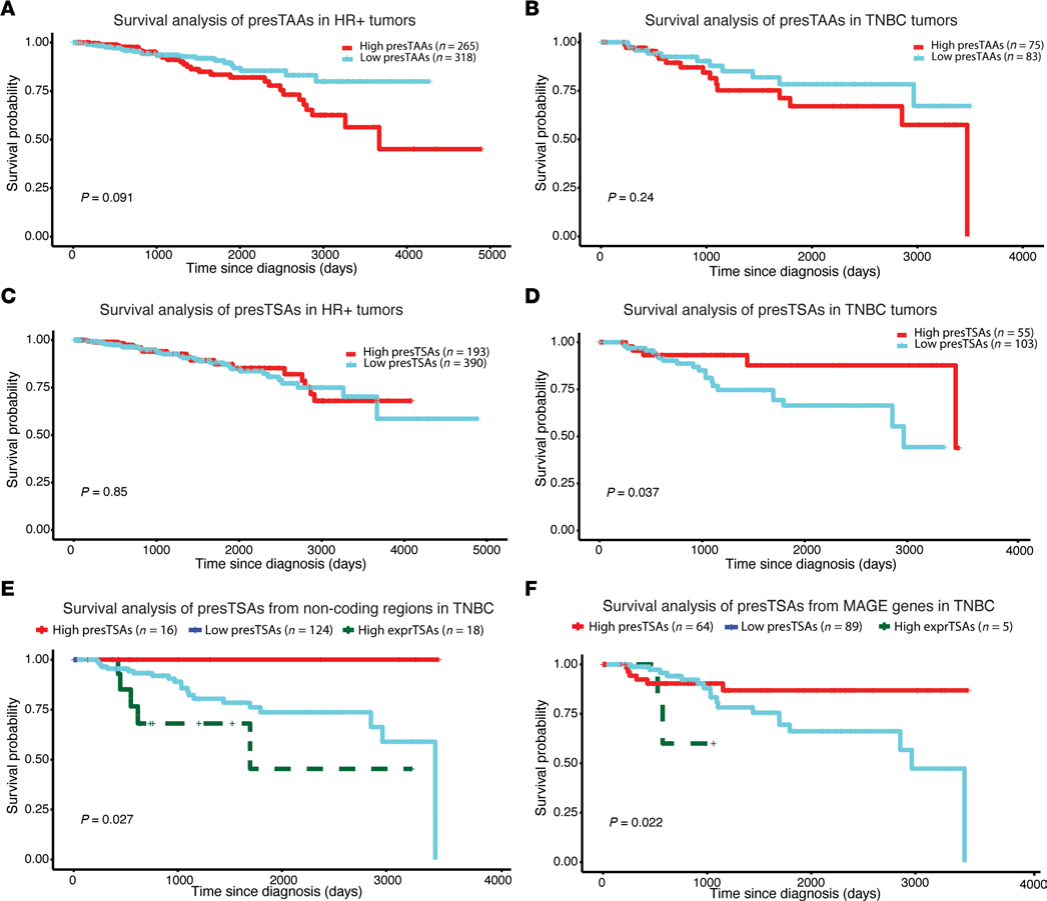
aeTSAs predicted presentation confers a survival advantage to patients with TNBC tumors. For each tumor, antigens were considered presented (presTSAs or presTAAs) if their coding sequence and an adequate HLA allele for the presentation were expressed. High groups (red) correspond to patients with the highest numbers of presTSAs or presTAAs (median). Low groups (turquoise) correspond to patients with lower numbers of presTSAs or presTAAs (median). (**A** and **B**) Survival analysis of predTAAs in HR+ and TNBC tumors. (**C** and **D**) Survival analysis of predTSAs in HR+ and TNBC tumors. (**E** and **F**) Survival analysis of predTSAs originating from noncoding regions or MAGE genes in TNBC tumors. To distinguish the impact of expression as opposed to the presentation of aeTSAs, an additional curve was computed with expressed antigens that lacked an appropriate HLA allele for presentation (exprTSAs).

The number of presented TAAs had no impact on the survival of patients with HR+ or TNBC tumors (Figure 7, A and B). Likewise, the number of presented aeTSAs had no effect in patients with HR+ tumors (Figure 7C), a group that expresses few aeTSAs (Figure 3C). However, the presentation of more numerous aeTSAs correlated with better overall survival in the TNBC cohort (Figure 7D). This benefit was observed with aeTSAs deriving from both MAGE genes and noncoding regions (Figure 7, E and F). Notably, this survival advantage was not reiterated when we considered only TSA expression (and not the HLA allotypes) (Figure 7, E and F). This means that the favorable impact of aeTSA presentation is HLA restricted. Hence, it is due to the MHC I presentation of the aeTSA peptide and not to the expression of the aeTSA-coding transcripts per se.

We conclude that aeTSAs reported herein confer an MHC I–restricted survival advantage in patients with TNBC (Figure 7D). This is not the case in patients with HR+ tumors (Figure 7C), probably because they present fewer aeTSAs than TNBC tumors (Figure 3C). Presentation of TAAs does not confer a similar survival advantage, likely because TAA expression is linked to activation of immunosuppressive pathways (Figure 4C).

## Discussion

The immunopeptidomes of TNBC and HR+ breast cancers display conspicuous similarities: source genes are highly overlapping, mTSAs are exceedingly rare, and TAAs are common in both. There is, however, one clear immunopeptidomic difference between TNBC and HR+ breast cancers: aeTSAs are frequently found in TNBCs, but rarely in HR+ tumors. Discrepancies in the number of aeTSAs provide a plausible molecular rationale for the prominent immunoreactivity of TNBC relative to HR+ breast cancers. Notably, TNBCs presenting numerous aeTSAs are associated with superior overall survival. Since this advantage is MHC I restricted, we can assume it depends on CD8^+^ T cells. Moreover, the number of presented aeTSAs also positively correlated with survival in acute myeloid leukemia (12). As aeTSAs show in vitro immunogenicity, they represent attractive targets for antigen-specific immunotherapies based on vaccines, TCR-containing biologics, and cell therapy. In contrast, the scarcity of mTSAs identified in our data highlights their low relevance as therapeutic tools in breast cancer. Indeed, although highly mutated genes did generate MAPs, they did not lead to mTSA generation. Two nonmutually exclusive hypotheses explain this: mutated regions are not located in MAP hotspots or are eliminated via immunoediting during tumor evolution (27, 48).

Our samples came exclusively from untreated patients. This study clearly shows that HR+ tumors from untreated patients rarely express aeTSAs. However, (a) expression of aeTSAs (e.g., from the MAGE family) can be enhanced by hypomethylating agents, and (b) one family of hypomethylating drugs (CDK4/6 inhibitors) is currently used as the first line of treatment in HR+ breast cancer. Hence, further studies are warranted to explore aeTSA expression in HR+ tumors treated with hypomethylating agents.

Both TNBC and HR+ tumors express numerous highly shared TAAs. Although most tested TAAs showed in vitro immunogenicity, they do not seem to elicit spontaneous antitumor responses, perhaps because their expression coincides with the proliferation of CAFs and activation of pathways instrumental in immune evasion (PI3K, WNT, MAPK) (49, 50). We propose that currently available inhibitors of these pathways could synergize with immune targeting of TAAs. Of relevance, PI3K inhibitors are approved for treating HR+ breast cancer and were shown to enhance effector CD8^+^ T cell activity in mouse breast cancer models (51).

One class of TAA may be particularly attractive for immune targeting: the 14 TAAs derived from CAFs. By creating extracellular matrix components, fibroblasts contribute to tumor growth and immune evasion (52). Therefore, strategies aiming at the depletion of CAFs or extracellular matrix components are actively investigated. However, antibody-based approaches have been fraught with several difficulties (53) and have not yet led to clinically meaningful responses in humans. Targeting CAF antigens with T cells might represent an effective strategy for depleting these tumor cell allies.

As a final comment, we wish to emphasize that there are commonalities and differences in the biogenesis of the immunopeptidome in different cancer types. Changes in methylation are a recurring theme (12, 15) and probably explain the expression of aeTSAs of the MAGE family in the present study. However, the cancer immunopeptidome also reflects key events specific to different cancer types: amplification of chromosome 3 in ovarian cancer (15), intron retention in acute myeloid leukemia (12), and extracellular matrix production in breast cancer (this study). Likewise, while most of the actionable antigens found in ovarian cancer and acute myeloid leukemia originate from non–protein-coding regions (12, 15), exons are their primary source in breast cancer. We should, therefore, be cautious in extrapolating immunopeptidomic concepts from one tumor type to another.

## Methods

### Primary breast cancer samples.

Fresh frozen primary breast tumor samples were bought from Tissue Solutions. Samples had a histological diagnosis of invasive ductal carcinoma (*n* = 24) or invasive carcinoma (*n* = 2). Immunohistochemistry data were available through Tissue Solutions, and samples were categorized as HR+ (ER or PR positive and HER2 negative; *n* = 14) or TNBC (ER/PR/HER2 negative; *n* = 12). Patients did not receive chemotherapy before resection (Supplemental Table 1).

### RNA and smRNA-Seq.

Total RNA was isolated using the All Prep DNA/RNA/miRNA Universal kit (QIAGEN) according to the manufacturer’s instructions. RNA was quantified using Qubit (Thermo Scientific), and quality was assessed with the 2100 Bioanalyzer (Agilent Technologies). Transcriptome libraries were generated using KAPA RNA HyperPrep (Roche) using a poly-A selection (Thermo Scientific). smRNA libraries were prepared using the QIAseq miRNA Library Kit (QIAGEN). Sequencing was performed on the Illumina NextSeq500 system (Supplemental Table 1).

### Database generation.

All RNA-Seq reads were trimmed by Trimmomatic, version 0.35, and aligned with STAR, version 2.5.1b, to the GRCh38 human genome using annotations from Ensembl release 99. Transcript expression was quantified in transcript per million (TPM) using Kallisto, version 0.43.0, with default parameters.

### Canonical proteomes.

Canonical proteomes were built as previously described (14). Sample-specific exomes were built using pyGeno (54) by inserting single-base variants (quality > 20) identified with FreeBayes (55). Annotated open-reading frames with TPM of greater than 0 were translated in silico from sample-specific exomes, creating the canonical proteome.

### ERE proteomes.

ERE proteomes were built for individual samples as described (34). Ambiguous nucleotides were trimmed from reads of the ERE data set, followed by a translation in all possible reading frames. Finally, the resulting ERE amino acid sequences were spliced to remove sequences following stop codons. Only sequences of at least 8 amino acids were kept and given a unique ID to generate a theoretical ERE proteome. This database was then concatenated to the canonical proteome, generating the personalized ERE proteome used for MAP identification.

### smRNA proteomes.

smRNA-Seq reads were concatenated in fastq.gz files. K-mer databases (24 long) were generated with Jellyfish, version 2.2.3, and assembled into contigs as previously described (12). Then we 3-frame translated the contigs and aggregated the various polypeptides into synthetic sequences of approximately 10k aa using the sequence JJ as a linker between the polypeptides. This manipulation was performed to avoid a bias in PEAKS linked to the use of a large number of shorter sequences. The obtained multi-fasta was concatenated with each sample’s canonical proteome for MAP identification.

### Cancer-specific proteomes.

Cancer-specific proteomes were assembled using k-mer profiling as described (12, 14). k-mers (33 nucleotides long) present at least twice in the mTECs k-mer database were removed from each cancer sample database, and the remaining k-mers were assembled into contigs. Finally, we 3-frame translated the contigs and linked the different polypeptides with JJ linkers. This database was concatenated with each sample’s canonical proteome for MAP identification.

### MS analyses.

MHC I immunoprecipitation, TMT labeling, and liquid chromatography-MS/MS analyses (LC-MS/MS) were performed as described (56). Of note, TMT labeling was used to improve the identification of peptides and was not used for quantification purposes (57). LC-MS/MS data were searched against the relevant database using PEAKS 10.5 or Peaks X Pro (Bioinformatics Solution Inc.). For peptide identification, tolerance was set at 10 ppm and 0.01 Da for precursor and fragment ions, respectively. Oxidation (M), deamidation, and TMT modification were set as variable modifications. Following peptide identification, we used the modified target-decoy approach built-in PEAKS to apply a sample-specific threshold on the PEAKS scores to ensure a FDR of 1%, calculated as the ratio between the number of decoy hits and the number of target hits above the score threshold. PEAKS scores corresponding to a 1% FDR for each sample were determined, and peptides that passed the threshold were further filtered to match the following criteria: peptide length between 8 and 11 amino acids and rank eluted ligand threshold of less than 2% based on NetMHCpan-4.1b (58). These filtering steps were performed with the use of MAPDP (59).

### Genomic origin and validation of MOIs.

The tumor antigen candidates were identified as previously described (12, 14, 15). When a MAP aligned to an exonic sequence with a 1 or greater read, the genomic origin of the MAP was considered exonic. Otherwise, the alignment with the highest number of reads was used to confer the genomic origin of the MOI. All final alignments for TSAs and TAAs were manually validated with Integrative Genomics Viewer (IGV) (https://igv.org/doc/desktop/). MOIs for which reads did not match a concordant genomic location or matched hypervariable regions (such as the MHC, Ig, or TCR genes) were excluded.

TSA candidates were classified as mTSAs if they contained variants in their MAP-coding sequences that did not match known germline polymorphisms reported in the Database of Single-Nucleotide Polymorphisms (dbSNP), version 149 (60).

Leucine and isoleucine variants are not distinguishable by standard MS approaches. Hence, we discarded MAPs for which an existing variant was flagged as non-MOI unless it presented a higher RNA expression than the variant or mapped in inadequate regions (in the case of ERE MAP candidates and smRNA MAP candidates). Additionally, all MOI, ERE MAP, and smRNA MAP candidates were validated with COMET software (2020.01 rev.4) (61) using the filters used with Peaks and an FDR of 5%. Manual spectra validation was done in-house for peptides not reidentified with COMET to further keep or discard MAPs. All possible gene IDs for MAP-deriving proteins reported in the HLA Ligand Atlas were obtained with BioMart (62).

### MOI expression in tissues.

As previously described by Ehx et al. (12), the expression of the MOI coding sequence was evaluated in normal tissues from (a) GTEx (50 normal tissues, 5–150 samples per tissue), (b) mTECs (*n* = 11), (c) purified blood and bone marrow samples (*n* = 4–16), and (d) breast cancer tissues from TCGA (*n* = 1,109) (Supplemental Table 2).

### Predicted TAA and TSA presentation in TCGA breast cancer samples and survival analyses.

A TAA or TSA was considered to be presented in a TCGA sample only when the MAP-coding transcript was highly expressed (>2 rphm and >nonnull median) and the patient had an HLA allotype that could present this MAP according to NetMHCPan4.1 (58). HLA alleles for TCGA patients were determined with Optitype software (v.1.3.5) (63). TCGA survival data were obtained with the TCGAbiolinks package (64). Patients with more than 1 biopsy were removed from the analyses so as not to duplicate their contribution to the results, leaving 915 patients. Kaplan-Meier survival curves and log-rank tests were generated with the TCGAbiolinks package.

### Single-cell analysis.

Aligned single-cell reads from 14 breast cancer patients published by Qian et al. (44) were obtained through their website (http://scope.lambrechtslab.org/). Visualization graphs were developed with the Seurat package (65).

### In silico immunogenicity prediction.

Immunogenicity predictions of MOI were performed with Repitope (66). Feature computation was performed with the predefined MHCI_Human_MinimumFeatureSet variable and FeatureDF_MHCI and FragmentLibrary files provided on the Mendeley repository of the package (https://data.mendeley.com/datasets/sydw5xnxpt/1).

### FEST assays.

FEST assays were conducted as described (67), with minor modifications. Briefly, on day 0, thawed peripheral blood mononuclear cell (PBMCs) from healthy donors (BioIVT) were T cell enriched using the Human Pan T Cell Isolation Kit (Miltenyi Biotec). Five million T cells were cocultured during 20 days, with a restimulation at day 10, with autologous T cell–depleted PBMCs pulsed with individual peptides. After in vitro expansion, CD8^+^ T cells were isolated using the Human CD8^+^ T Cell Isolation Kit (Miltenyi Biotec). DNA extraction was performed with a QIAGEN DNA Blood Mini Kit and was followed by TCR Vβ CDR3 sequencing using the ImmunoSEQ platform. Raw data were processed with the FEST web tool (www.stat-apps.onc.jhmi.edu/FEST). The following parameters were used: FDR 1%, fold change >5, minimal number of templates of 1, and “Ignore baseline threshold.” Our 2 negative control groups consisted of CD8^+^ T cells cocultured with unpulsed autologous T cell–depleted PBMCs and uncultured CD8^+^ T cells.

### GSEA.

GSEAs were performed with the fgsea package (68). These analyses were performed using RNA-Seq data from TCGA. First, we divided samples presenting high (above median) versus low (below median) numbers of aeTSAs or TAAs. TAAs or aeTSAs were considered to be presented in a TCGA sample when their MAP-coding transcripts were expressed (> rphm) and the patient had an HLA allotype that could present this MAP according to NetMHCPan4.1 (58). Then we performed a differential gene expression analysis between the high and low groups. These analyses were performed on all TCGA samples or separately in HR+ and TNBC samples, as indicated in the main text. Using known lists from Danaher et al. (39) and the Molecular Signature Database, we could describe different enrichments presented in Figure 3D, Figure 4C, and Supplemental Figure 4, B and C. For GSEAs, TAAs or TSAs were considered to be presented in a TCGA sample when their MAP-coding transcripts were expressed (>2 rphm) and the patient had an HLA allotype that could present this MAP according to NetMHCPan4.1.

### Data and materials availability.

MS raw data and PEAKS searches were deposited to the ProteomeXchange Consortium via the PRIDE partner repository with the following data set identifiers: PXD034818. RNA-Seq data were deposited in the NCBI’s Sequence Read Archive (SRA) (PRJNA852282) and Gene Expression Omnibus database (GEO GSE206838) (Supplemental Table 1). Values for all data points in graphs are reported in the Supporting Data Values file.

### Statistics.

Analyses and figures were performed using R, version 4.0.0. The gplots and ggplot2 packages in R were used to generate the different graphs. Tests involving comparisons of distributions were performed with the *t* test or the 1-way ANOVA test, as necessary. Differential gene expression analyses were performed with the limma package (69). GSEAs were performed with the fgsea package (68). Kaplan-Meier survival curves and log-rank tests were generated with the TCGAbiolinks package. Significant *P* values are indicated in each legend.

### Study approval.

This project was approved by the Clinical Research Ethics Committee from the University of Montreal (CERC-20-012-D).

## Author contributions

EK, PT, and CP conceived and designed the project. EK, MPH, KV, AA, QZ, JPL, CD, JL, MC, and JDL developed methodology. EK, JPL, CD, MC, MPH, KV, LH, and CT acquired data (provided animals, acquired samples, provided facilities, etc.). EK, JPL, CD, MC, QZ, AA, JDL, MVRC, MPH, KV, LH, and CT analyzed and interpreted data (e.g., statistical analysis, biostatistics, computational analysis). EK, JPL, CD, JL, MC, QZ, AA, JDL, MPH, KV, PG, LH, CT, MVRC, GE, PT, and CP wrote, reviewed, and/or revised the manuscript. JPL, PG, MC, MPH, KV, LH, and CT provided administrative, technical, or material support (i.e., reporting or organizing data, constructing databases). PT and CP supervised the study.

## Supplementary Material

Supplemental data

Supplemental table 1

Supplemental table 2

Supplemental table 3

Supplemental table 4

Supplemental table 5

Supporting data values

## Version 1 10/31/2023

In-Press Preview

## Version 2 01/02/2024

Electronic publication
