# Supplementary material for: Breast cancer immunopeptidomes contain numerous shared tumor antigens
Source: J Clin Invest. 2024 Jan 2;134(1):e166740. doi: 10.1172/JCI166740 (PMC10760959; doi:10.1172/JCI166740)
Supplement: Supplemental data [file jci-134-166740-s036.pdf]

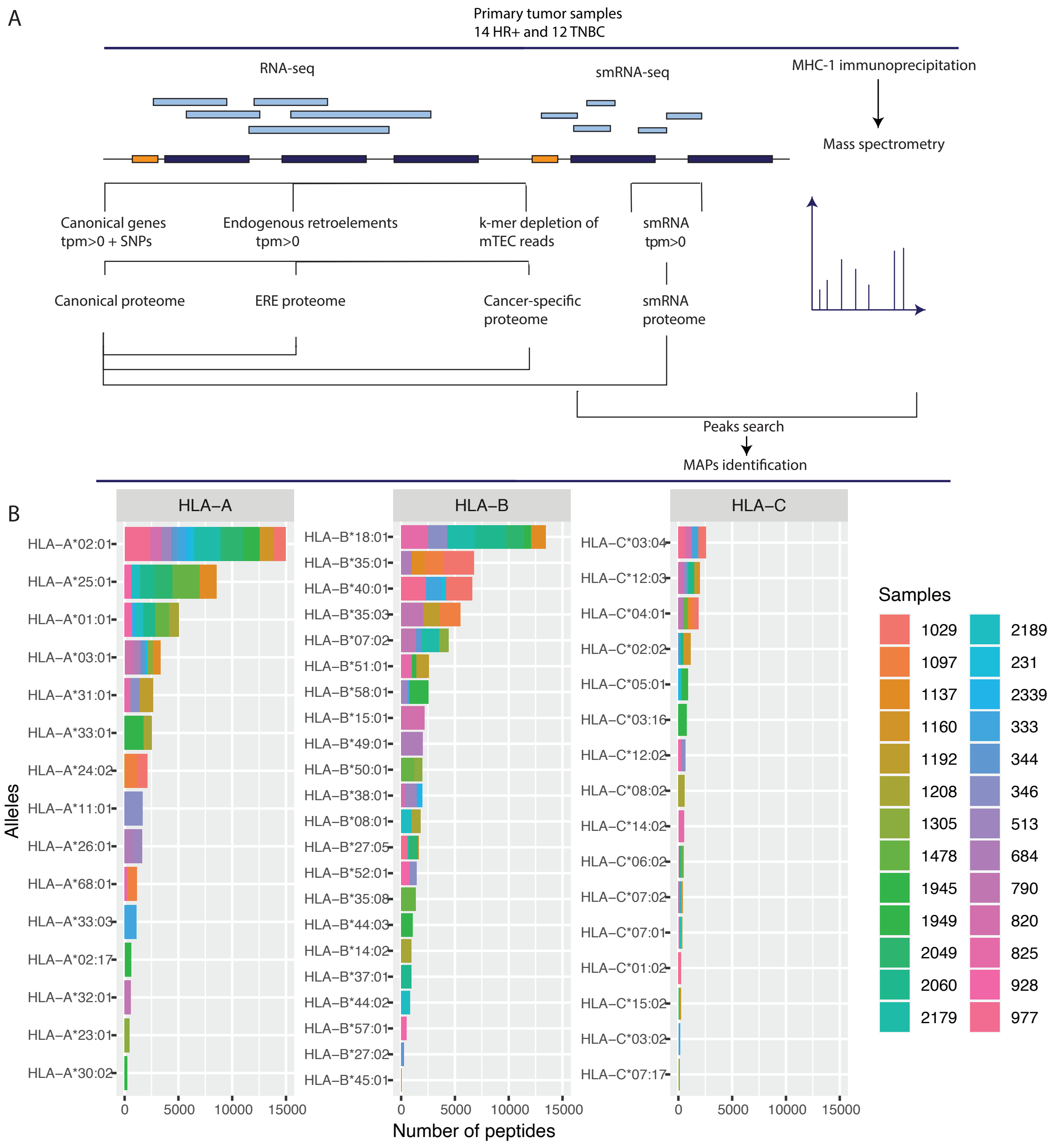

Supplementary figure 1. (A) Illustration of the database constructs used for Peaks searches. Personalized databases are basez on a canonical proteome, with the addition of an ERE proteome, a small-RNA (smRNA) proteome or a cancer-specific proteome. (B) Number of MAPs identified for each HLA allele (n=53) in our dataset (n = 26).

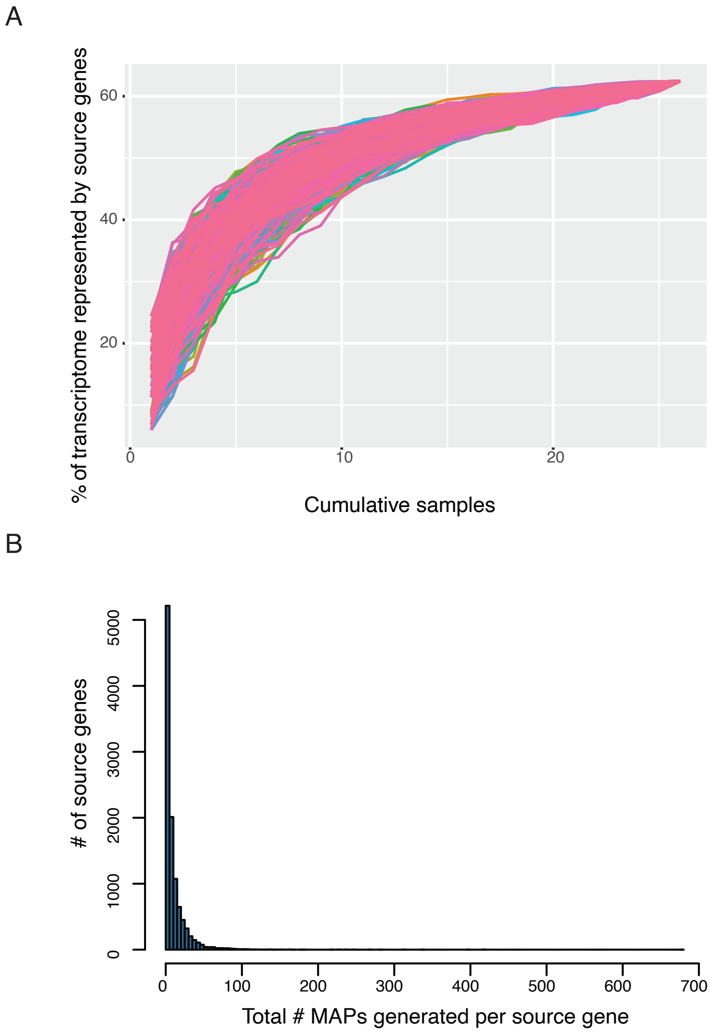

Supplementary figure 2.
A) Percentage of the transcriptome represented at the immunopeptidomic level per cumulative samples. Multiple random iterations (n=500) are illustrated in regard to the order of the addition of samples.
B) Histogram of the total number of MAPs generated per source gene in our dataset.

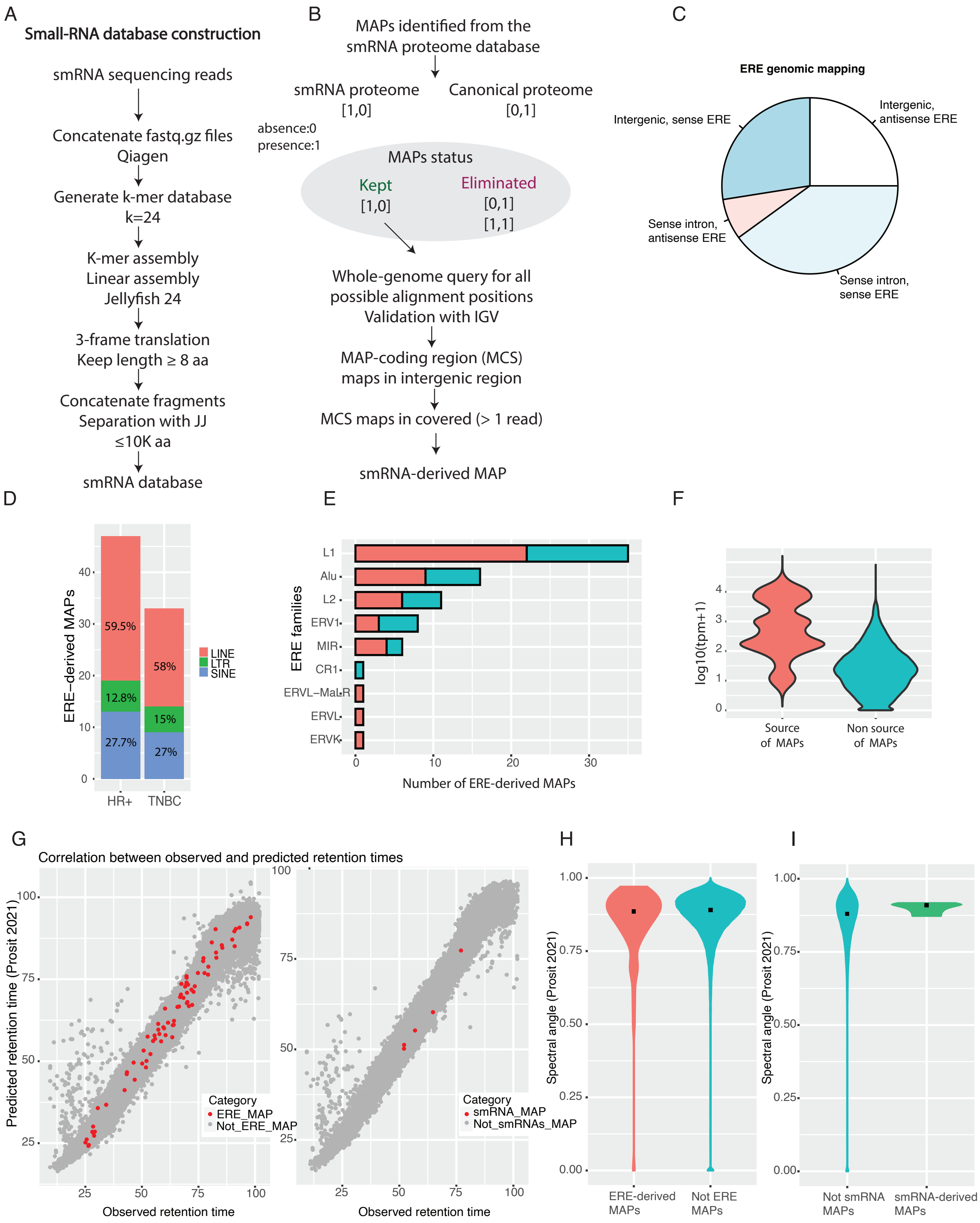

Supplementary figure 3.

(A) Small-RNA database construction workflow. (B) Filtering steps for peptide annotation and validation for smRNA-derived MAPs. (C) Genomic mapping of ERE-derived MAPs. (D) Number of ERE-derived MAPs per ERE class. (E) Number of MAPs identified per ERE family in HR+ (red) and TNBC (blue) samples. (F) Expression level of ERE subfamilies that generate or not MAPs (t-test; p<0.001). (G) Pearson correlation between predicted and observed retention times for ERE- (r = 0.98, p<0.001) and smRNA- derived MAPs (r = 0.98, p<0.001). (H) Distribution of spectral angles for ERE-derived MAPs (mean = 0.85 vs 0.86, f-test = 0.15). (I) Distribution of spectral angles for smRNA- derived MAPs (mean = 0.84 vs 0.9, f-test =0.001).

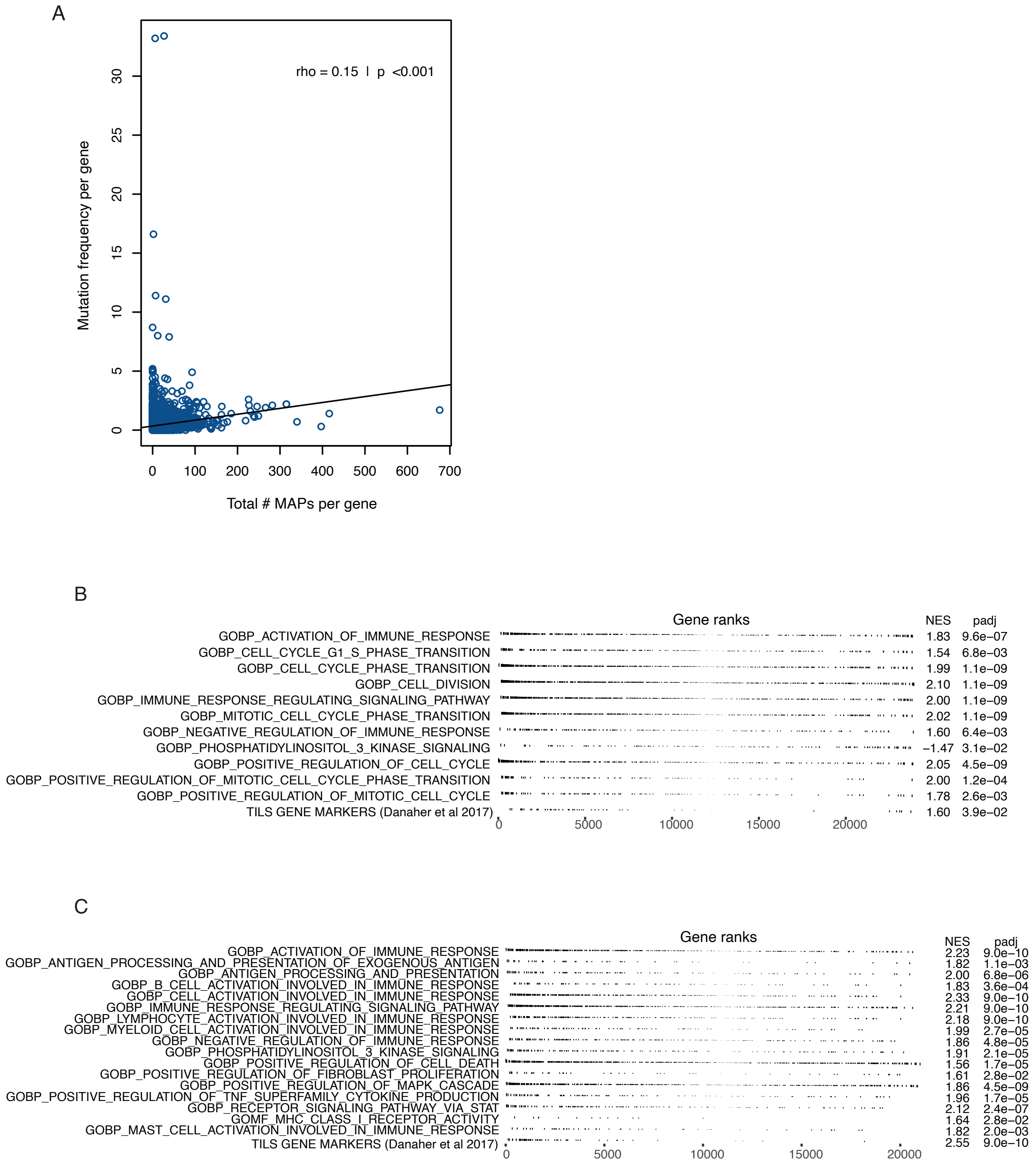

Supplementary figure 4.
(A) Spearman correlation between the number of MAPs identified per gene in our dataset and their frequency of mutations (as described in the breast cancer TCGA cohort). (B) GSEA enrichment analysis in all breast cancer tumors from TCGA (n = 1109) with high levels (> median) of predicted TSAs (as defined by : expression > 2 rphm and adequate HLA allele for presentation). (C) GSEA enrichment analysis in TNBC tumors from TCGA (n=158) with high levels (> median) of predicted TAAs (as defined by : expression > 2 rphm and adequate HLA allele for presentation).
